# Birth Order, Caesarean Section, or Daycare Attendance in Relation to Child- and Adult-Onset Type 1 Diabetes: Results from the German National Cohort

**DOI:** 10.3390/ijerph191710880

**Published:** 2022-08-31

**Authors:** Justine Tanoey, Christina Baechle, Hermann Brenner, Andreas Deckert, Julia Fricke, Kathrin Günther, André Karch, Thomas Keil, Alexander Kluttig, Michael Leitzmann, Rafael Mikolajczyk, Nadia Obi, Tobias Pischon, Tamara Schikowski, Sabine M. Schipf, Matthias B. Schulze, Anja Sedlmeier, Ilais Moreno Velásquez, Katharina S. Weber, Henry Völzke, Wolfgang Ahrens, Sylvia Gastell, Bernd Holleczek, Karl-Heinz Jöckel, Verena Katzke, Wolfgang Lieb, Karin B. Michels, Börge Schmidt, Henning Teismann, Heiko Becher

**Affiliations:** 1 Institute of Medical Biometry and Epidemiology, University Medical Center Hamburg-Eppendorf, 20246 Hamburg, Germany; 2 Institute for Biometrics and Epidemiology, German Diabetes Center (DDZ), Leibniz Institute for Diabetes Research, Heinrich Heine University, 40225 Düsseldorf, Germany; 3 Clinical Epidemiology and Aging Research, German Cancer Research Center (DKFZ), 69120 Heidelberg, Germany; 4 Heidelberg Institute of Global Health, Heidelberg University Hospital, 69120 Heidelberg, Germany; 5 Institute of Social Medicine, Epidemiology and Health Economics, Charité—Universitätsmedizin Berlin, 10117 Berlin, Germany; 6 Leibniz Institute for Prevention Research and Epidemiology—BIPS, 28359 Bremen, Germany; 7 Institute for Epidemiology and Social Medicine, Albert-Schweitzer-Campus 1, Building D3, 48149 Münster, Germany; 8 Institute of Clinical Epidemiology and Biometry, University of Würzburg, 97080 Würzburg, Germany; 9 State Institute of Health, Bavarian Health and Food Safety Authority, 91058 Erlangen, Germany; 10 Institute for Medical Epidemiology, Biometrics and Informatics, Interdisciplinary Center for Health Sciences, Martin Luther University Halle-Wittenberg, 06112 Halle (Saale), Germany; 11 Department for Epidemiology and Preventive Medicine, Regensburg University Medical Center, 93053 Regensburg, Germany; 12 Max-Delbrück-Center for Molecular Medicine in the Helmholtz Association, Molecular Epidemiology Research Group, 13125 Berlin, Germany; 13 Max-Delbrück-Center for Molecular Medicine in the Helmholtz Association, Biobank Technology Platform, 13125 Berlin, Germany; 14 Charité—Universitätsmedizin Berlin, Freie Universität Berlin and Humboldt-Universität zu Berlin, 10117 Berlin, Germany; 15 Leibniz Research Institute for Environmental Medicine—IUF, 40225 Düsseldorf, Germany; 16 Institute for Community Medicine, University Medicine Greifswald, 17489 Greifswald, Germany; 17 German Institute of Human Nutrition Potsdam-Rehbruecke, 14558 Nuthetal, Germany; 18 Institute of Nutritional Science, University of Potsdam, 14558 Nuthetal, Germany; 19 Institute of Epidemiology, Kiel University, 24105 Kiel, Germany; 20 Institute of Medical Informatics, Biometry und Epidemiology, Essen University Hospital, 45147 Essen, Germany; 21 Division of Cancer Epidemiology, German Cancer Research Center (DKFZ), 69120 Heidelberg, Germany; 22 Institute for Prevention and Cancer Epidemiology, Faculty of Medicine and Medical Center, University of Freiburg, 79110 Freiburg, Germany

**Keywords:** perinatal, adult-onset, late-onset, autoimmune, delivery mode, sex, offspring, NAKO

## Abstract

(1) Background: Global incidence of type 1 diabetes (T1D) is rising and nearly half occurred in adults. However, it is unclear if certain early-life childhood T1D risk factors were also associated with adult-onset T1D. This study aimed to assess associations between birth order, delivery mode or daycare attendance and type 1 diabetes (T1D) risk in a population-based cohort and whether these were similar for childhood- and adult-onset T1D (cut-off age 15); (2) Methods: Data were obtained from the German National Cohort (NAKO Gesundheitsstudie) baseline assessment. Self-reported diabetes was classified as T1D if: diagnosis age ≤ 40 years and has been receiving insulin treatment since less than one year after diagnosis. Cox regression was applied for T1D risk analysis; (3) Results: Analyses included 101,411 participants (100 childhood- and 271 adult-onset T1D cases). Compared to “only-children”, HRs for second- or later-born individuals were 0.70 (95% CI = 0.50–0.96) and 0.65 (95% CI = 0.45–0.94), respectively, regardless of parental diabetes, migration background, birth year and perinatal factors. In further analyses, higher birth order reduced T1D risk in children and adults born in recent decades. Caesarean section and daycare attendance showed no clear associations with T1D risk; (4) Conclusions: Birth order should be considered in both children and adults’ T1D risk assessment for early detection.

## 1. Introduction

Living with type 1 diabetes (T1D) requires lifelong self-management of regular insulin intake and blood glucose monitoring, while maintaining a generally healthy lifestyle. Since it is a chronic autoimmune disease, continuous proper care is mandatory to prevent acute complications of hypoglycemia and diabetic ketoacidosis, attenuate chronic complications or impede death [1]. It is a challenging condition not only for the individual, but also the families, as T1D is typically diagnosed in childhood [2]. Moreover, younger T1D onset age corresponds to higher cardiovascular disease morbidity and all-cause mortality risk [3]. As one of the most common endocrine and metabolic diseases in childhood, over 600,000 children under age 15 were estimated to live with T1D worldwide in 2019, with more than 120,000 new cases diagnosed each year [4]. Adult-onset T1D is more difficult to clinically differentiate from type 2 diabetes (T2D) and previously believed to be less prevalent than in children [5,6]. However, recent studies have reported up to more than half of all new T1D diagnoses occurred in adults [7,8].

Genetic predispositions that are more frequent in certain ethnicities are evident risk factors for T1D [9,10]. However, genetics alone could not explain the discordant T1D diagnoses seen in monozygotic twins and the rising incidence rates in recent decades [11,12,13]. This enigma has led to the research of non-genetic factors that might contribute to autoimmune seroconversion and progression to T1D [9,14].

One driving theory for these factors was the role of the human intestine and the microflora colonizing the organ as part of the immune system. However, emerging evidence from studies among school-aged children indicated that the maturation rates of gut microbiome towards adults’ composition might be slower than previously reported, and, hence, the “window of opportunity” for external influences was longer [15]. In addition, these rates might also have consequences on the functionality of the immune system that extends beyond childhood [16,17]. Although direct causality has not been proven, few studies investigating gut microbiome in children with T1D risk have noted less diversity (dysbiosis) and composition differences in those who developed pancreatic beta-cell autoantibodies [18,19].

Children delivered by Caesarean section (C-section) have been shown to have gut microbiome dysbiosis and altered immune function relative to those delivered vaginally [20,21,22,23,24]. These findings suggest C-section could be a proxy to investigate T1D risk associated with adverse gut microbiome. Moreover, C-section has become quite a common procedure in recent decades, with 1 in 3–5 births in Europe, including Germany, performed by surgery [25,26], calling for rigorous assessment into its potential long-term effects. A pooled meta-analysis of 20 observational studies found an increased T1D risk attributable to C-section deliveries [27]. However, few studies into this association included T1D individuals diagnosed beyond adolescent years [27,28,29,30,31]. Additionally, while many studies were able to adjust for maternal diabetes, paternal diabetes, an essential risk factor for T1D in the offspring, was often unaccounted for in the analyses [27,28,29,30,31].

Exposure to certain pathogens has also been discussed as necessary in healthy immune system development, which in turn might assist in mitigating T1D risk [32]. Since accurate recollection of all childhood infections that could shape the gut microbiome development was unlikely, surrogate indicators such as birth order or daycare attendance have been applied [32,33,34,35,36]. Epidemiological studies including a large meta-analysis have reported reduced T1D risk in children and adolescents who have older siblings [37,38,39]. In contrast, only a single study was found on older individuals (aged 15–39 years), and it did not find a similar effect [40].

In addition, incidence rates for childhood-onset T1D showed no obvious sex dominance, in contrast to higher incidence rates among adult males reported by various studies [41,42]. This observation, supported by evidence that sex might well play a role in pancreatic islet cell preservation and autoimmunity, particularly after adolescence [43,44], also prompted this investigation comparing the associations between early childhood risk factors and T1D risk among childhood- and adult-onset cases.

There is sparse evidence on the impact of mode of delivery, birth order, and attending daycare on T1D risk in individuals beyond age 15 years in the general population. Therefore, the objectives of this study were to assess (i) the associations between the above-mentioned factors and risk of likely T1D in a population-based cohort and (ii) whether the associations were similar for childhood- and adult-onset diabetes.

## 2. Materials and Methods

### 2.1. Data Source and Study Population

The German National Cohort (NAKO Gesundheitsstudie) is a large prospective cohort study in Germany. Initial recruitment in 18 regional centers took place from 2014 to 2019 and has included 205,000 male and female individuals aged 20–69 years. A detailed description of the study has been published [45] and is also available on its website https://nako.de/ (accessed on 2 September 2019) [46]. Selected data regarding migration history, demographic information, own and parental history of diabetes, as well as childhood health information, were part of the interviews and self-completed questionnaires. We used data from 101,628 participants from the NAKO, which represented the first half of the recruited individuals.

### 2.2. Type 1 Diabetes Ascertainment

Diabetes diagnosis and age at diagnosis in the study originated from self-report according to the questions “Have you ever been diagnosed with diabetes by a physician” and, if yes, “In what year or at what age was diabetes first diagnosed?” In addition, the kind of therapy and age or year when insulin treatment was initiated were attained.

The algorithm used to classify diabetes type was preliminarily based on criteria recommended by the WHO. Since these were not clearly cut with respect to age at diagnosis, we additionally applied those of the UK’s RCGP (Royal College of General Practitioners) and previous studies assessing clinical characteristics of T1D patients and optimal T1D classification algorithms based on clinical features [47,48,49,50,51]. A cohort member with self-reported diabetes was therefore classified as T1D under the following conditions:

- Age at diagnosis ≤40 years.
- Ongoing insulin therapy at time of recruitment.
- Time from diagnosis to insulin therapy initiation ≤1 year.

Individuals with missing information on insulin therapy but age at diagnosis ≤40 years were also considered as T1D in the main analyses, unless the first diagnosis was made during pregnancy. All other individuals reporting a diabetes diagnosis were considered as having type 2 or other types of diabetes.

In previous validation studies [50], the above algorithm showed 76–97% sensitivity and 59–97% specificity from diagnosis age or 92–100% sensitivity and 75–82% specificity from insulin therapy beginning in less than 2 years of diagnosis. Combining all criteria should discern type 1 and type 2 diabetes in approximately 90% of cases.

### 2.3. Exposure Variables and Covariables

Exposure variables of main interest were birth order, daycare attendance and C-section delivery. The variable birth order was grouped into only child (not including half or step siblings), first, second and third or more. The variables daycare attendance (enrollment at age 0–6 years) and C-section delivery were categorized as yes or no.

Based on prior publications, the following covariables were considered as possible confounders: paternal and maternal diabetes, migration background, birth year and self-reported perinatal factors (prematurity, birth weight and being breastfed). Parental diabetes was grouped into no diabetes, known diabetes before age 40 and known diabetes after age 40 or unknown age. The variable migration background was derived from information on nationality, country of birth and parental country of birth. Self-reported weight at age 18 was used to estimate BMI at that age, which was categorized according to the WHO nutritional status [52]. Missing values for all variables except migration background and BMI at age 18 were categorized as “unknown”. Observations with missing values in migration background were excluded from Cox regression analyses, and only available BMI at age 18 values were applied in an adapted type 1 diabetes ascertainment algorithm for sensitivity analyses.

### 2.4. Statistical Analysis

Variables were analyzed descriptively and presented by diabetes category. Diabetes diagnosis age and therapy among participants with likely T1D by sex were laid out graphically. To investigate the association between potential risk factors and T1D risk, the variables were first analyzed in univariable Cox regression, stratified by birth year. An event was defined as likely T1D diagnosis based on our algorithm. Observation time was from birth until an event occurred or the age at recruitment, and age 40 years for non-events. Participants with diabetes diagnosis categorized as T2D or others were considered as non-events.

Exposure variables were subsequently analyzed in a multivariable Cox regression model, stratified by birth year and adjusted for paternal and maternal diabetes, migration background, prematurity, birth weight and being breastfed (full model), and for only paternal and maternal diabetes as well as migration background (reduced model). To investigate differential effects in males and females, separate analyses by sex were also performed.

To investigate the associations between the exposure variables and childhood- or adult-onset T1D, the events were stratified by diagnosis age (cut-off age = 15 years). In the analysis with childhood-onset T1D (event), likely T1D cases diagnosed at age > 15 were considered non-events, and observation time began at birth and ended at diagnosis age for events or age 15 for non-events. In adult-onset T1D analysis, likely childhood T1D cases were removed, and observation time began at birth and ended at diagnosis for events or between 20 and 40 years for non-events. Since full and reduced models demonstrated similar estimates, the same uni- and reduced multivariable Cox regression models were applied for the respective sub-cohorts.

For sensitivity analyses, the cohort with (i) all participants with type 2 or other types of diabetes (participants who reported diabetes but did not fit T1D classification criteria) removed, (ii) an alternative T1D identification criteria applied, and (iii) grouping into two sub-cohorts by birth year (cut-off year 1965), were analyzed. In (ii), the algorithm to determine diabetes type 1 was adapted as follows: (1) diagnosis age ≤ 50 years (later onset age), (2) not first detected during pregnancy, (3) treated only with insulin within one year of diagnosis (stricter therapy criterion) and (4) if therapy was unknown, then BMI at age 18 was <30 kg/m^2^ (not obese).

## 3. Results

**Figure 1:**
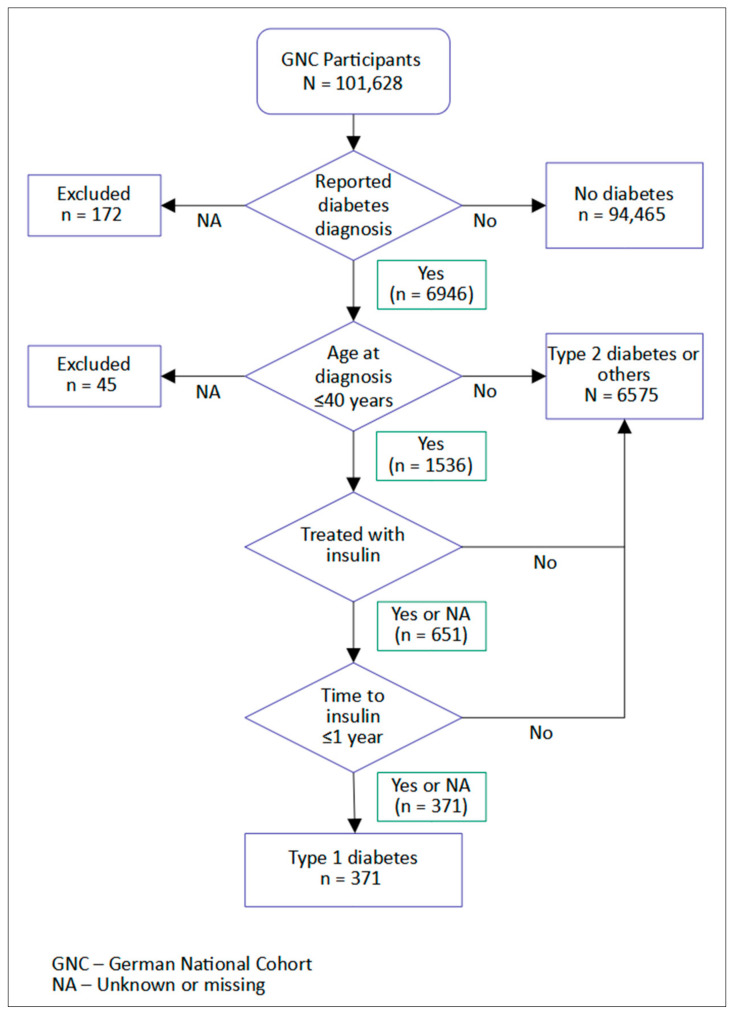
German National Cohort (GNC) type 1 diabetes case identification algorithm based on age at diagnosis and treatment with insulin.

From 101,628 participants, 217 were excluded as they did not respond to the question on diabetes history or provide age at diagnosis. The final cohort comprised 101,411 participants, of which 46.4% were male. The diabetes classification algorithm yielded 371 (0.4%) individuals with likely T1D, 6575 (6.5%) cases with likely T2D or other types and 94,465 individuals without diabetes (Figure 1).

**Figure 2:**
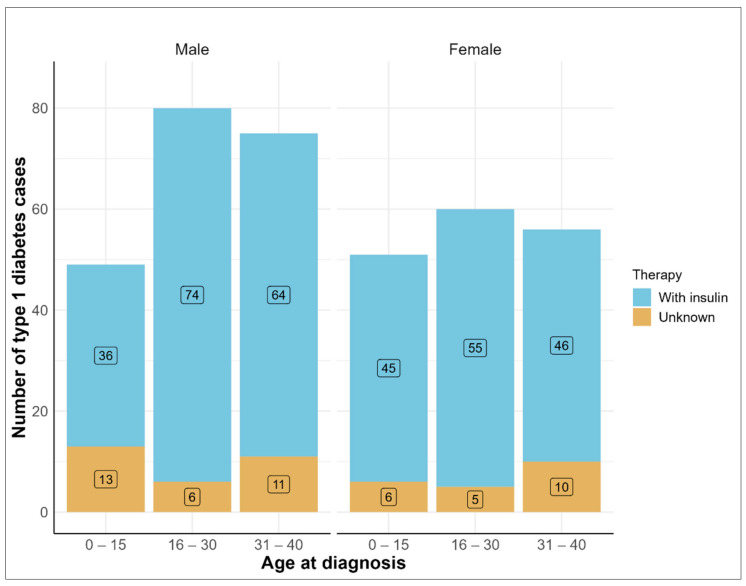
Age at diagnosis and insulin therapy by sex among the German National Cohort participants with type 1 diabetes.

There were 204 (57.6%) males and 167 (45.0%) females among the participants with T1D, with an overall mean age at diagnosis of 23.6 years. Detailed description of migration background, diabetes therapy, parental diabetes history, and perinatal and potential risk factors are presented in Table 1. The number of incident T1D cases increased slightly across the age groups, but more pronounced among males aged > 15 years (Figure 2).

Risk estimates from uni- and multivariable Cox regression analyses are shown in Table 2. Lower T1D risk was seen in females than males and individuals with higher birth orders compared to only children. Delivery by C-section estimates indicated possible increased T1D risk, but the confidence intervals included the null. Daycare attendance was not significantly associated with the risk of T1D. Further analyses revealed diagnosis age-related associations between birth order and T1D risk. In multivariable models adjusted for parental diabetes and migration background, the reduced risks for second- or later-born individuals was only significant for T1D diagnosed in childhood (onset age 0–15 years), but not in adulthood. In contrast, lower T1D risk for females was apparent in those diagnosed with T1D after age 15. In additional analyses, males and females were analyzed separately using the regression models from Table 2. Due to the lower sample sizes, the estimates show larger variation; however, the overall results were similar in both sexes (Supplementary Table S1).

Results from sensitivity analyses (Supplementary Table S2), where diabetes type 2 or other types (n = 6575) were removed (i), were similar to the main analysis. Significant risk reduction for females and second- or later-born individuals, and no association with C-section delivery or attending daycare were observed in all age multivariable analyses. Separate analyses for childhood and adult T1D risks demonstrated lower adult-onset T1D risk for females and lower childhood T1D risk for higher order-born individuals. Adapted T1D classification algorithm with older diagnosis age limit (up to 50 years) and either a sole treatment with insulin within 1 year of diagnosis or non-obese BMI at age 18 for the second sensitivity analyses (ii), identified 423 T1D cases. Analyses with this algorithm also yielded comparable risk estimates to the main analyses, although the childhood T1D risk reduction for higher birth-order individuals was less pronounced. Grouping with cut-off year 1965 (iii, Supplementary Table S3) showed that among those born after 1965, higher birth order was associated with reduced childhood- and adult-onset T1D risk and C-section was associated with higher overall T1D risk. Daycare attendance history was not associated with T1D risk, but reduced T1D risk among females was consistently seen in both groups. There was a higher proportion of unknown values across all variables of interest among earlier-born participants.

## 4. Discussion

To our knowledge, this was the first study that assessed the associations between birth order, delivery mode as well as daycare attendance and T1D risk, with cases in not only children, but also adults beyond young adulthood. Estimates from all-case analyses showed that individuals born second or later had lower risks of T1D, particularly in contrast to individuals without siblings. Age-at-diagnosis stratification revealed that the lower risks observed among higher-order-born individuals were more apparent in terms of childhood-onset T1D, but sub-cohort analyses by birth year demonstrated similar lower T1D risks for both childhood- and adult-onset T1D for second-born children and adults among those born in more recent decades. C-section delivery and daycare attendance did not show clear associations with T1D risk, although the estimates for C-section delivery pointed towards an increased risk for T1D onset in childhood.

In line with previous studies [37,39], our estimates revealed reduced T1D risks among individuals born with more older siblings. As children, such individuals might be exposed to a more diverse microbiome environment and earlier than only children or first-borns. This immediate postnatal exposure may benefit infants’ immune system maturation through infection of certain pathogens that might protect against autoimmune diabetes [32] and healthy gut microbiome colonization [20,33]. Indeed, numerous studies have reported reduced diversity, altered composition and function of the gut microbiome in children with T1D [32,53,54]. Further, we found that the effect was not evident in adult-onset T1D, concurrent with results from another study on T1D perinatal risk factors in young adults [40]. This age disparity could indicate that the underlying reasons or mechanisms were indeed more consequential at very young ages. It has also been observed that the protective effects of being born later decreased after age 5 [37]. Interestingly, lower risks for both childhood- and adult-onset T1D cases among those born after 1965 could suggest that any mechanisms behind this association were pertinent to relatively younger adults and not limited to childhood and adolescence. Concurrently, the effect of early childhood external exposures on gut microbiome establishment might a have prolonged impact on the immune system [15,16,17].

C-section was not clearly found to pose significant risk in our cohort after accounting for other factors. It is possible that any effect of C-section on T1D risk was essentially too small relative to genetics, since we controlled for both parents’ diabetes history, and as it has also been revealed in a sibling-design study [55], other environmental factors. Although we observed an increased risk associated with T1D in those born after 1965, this might be biased by the higher proportion of missing birth history among the older participants. The main postulated mechanism on how C-section might increase T1D risk in the offspring was through gut microbiome dysbiosis and dysfunction [22,56,57]. However, several studies have also reported that the impact ultimately declined beyond the first year of life, and additionally, other external factors such as breastfeeding could assist in rectifying the imbalance [23,33,56,58]. Another disadvantage of C-section on the offspring’s immune system development may be the lack of labor process in pre-labor (elective) C-section [59,60]. Results from a meta-analysis distinguishing the effects of pre-labor and emergency C-section compared to vaginal delivery suggested that there might be a slightly increased T1D risk from pre-labor C-section. However, heterogeneity between studies existed [28], and we were not able to explore this aspect due to data unavailability in the cohort.

Upon inclusion of other factors, also history of daycare attendance was neither associated with T1D risk in children nor adults in this study. Attending daycare indeed exposes very young children to abundant microbiome [36], but likely coincidentally to both commensal and pathologic infections. Consequently, any protective versus harmful effects might mask each other or be irrelevant in light of earlier exposures through same-household siblings or pets [33]. It is also possible that the effect was not seen as it was dependent on other factors or only relevant in very young children. Studies conducted elsewhere stated that the effect of daycare attendance was only apparent in conjunction with breastfeeding [35] or that risk reductions were observed in children under 5 [34].

Differences between childhood- and adult-onset T1D may be attributed to lower genetic risks and less presence of multiple diabetes-associated autoantibodies in adults [7], suggesting that other factors could contribute more to the disease manifestation. Progression to symptomatic diabetes is also slower in adults, and individuals with adult-onset T1D have more remaining functional pancreatic β-cells at diagnosis than children [7,61]. Additionally, our analyses indicated that there was no difference associated with sex in childhood-onset T1D, though adolescent years might be a turning point. It has been suggested that hormonal changes during puberty resulted in the presence of “17β-estradiol/E2”, an ovarian hormone that appeared favorable in preserving pancreatic islet cells from metabolic injuries. Estradiol also promotes immune tolerance that protects against autoimmune pancreatic islet destruction, which was also seen following Coxsackie virus infection [44], a virus that has been implicated in T1D progression [62,63]. Although the relationship between sex and T1D is complex, our findings together with prior studies underline its possible importance in diagnosis and therapy [44,64].

With regard to our findings on the role of birth order, C-section and daycare attendance on childhood T1D risk in our cohort, it must be kept in mind that these factors were surrogates for direct measurements of microbiome exposures, including infections. The role of gut microbiome on T1D progression itself in humans is yet to be proven, as it has not been determined whether the seemingly “unideal” composition was the cause or consequence of the disease. It is also still unclear which infections were protective or harmful, and to what extent [32]. It is plausible as well that the association is related to an individual’s genetic predisposition [62]. Moreover, the thymus gland is fundamentally responsible in educating T lymphocytes to tolerate insulin and other T1D-related antigens by releasing cells that tolerate the antigens (positive selection) and destroying cells that are auto-reactive (negative selection). Although a dysfunctional thymus alone does not cause T1D, and environmental factors such as viral infections and insulin resistance in obesity have been suggested to trigger disease progression, it is a necessary condition in T1D [63,65]. As thymus development begins early in pregnancy, it should be noted that exposures in fetal life such as maternal diabetes, maternal obesity, gestational age and congenital infections that could affect thymus growth and fetal immune development are of importance [65,66,67].

There are limitations in our study that need to be addressed. Our primary limitation was that this cohort study was not specifically designed to investigate T1D risk in the offspring. This created a challenge in identifying T1D cases retrospectively based on self-reported data, instead of gold-standard methods such as C-peptide or autoantibodies measurements. We applied an algorithm that has been reported to be fairly accurate in the main analysis, but it is possible that some cases were misclassified, particularly in participants only fitting the diagnosis age criterion and adults whose clinical features could be atypical of T1D [7,51]. Nonetheless, our findings were consistent in all applied sensitivity analyses, adding confidence in our results. Another challenge was that maternal characteristics, namely maternal age at birth [68], maternal obesity [69,70], and extended-family genetic risk [55,71], which are possible confounders, were not available in our dataset. Nevertheless, we were able to account for not only maternal but also paternal history of diabetes, two major indicators of T1D genetic predispositions [71]. Additionally, our data on daycare attendance did not allow the exact timing (age) of when the exposure began as the collected information was provided in age categories. However, only 11 out of 354 events might have been diagnosed with diabetes prior to beginning daycare, and excluding them did not change our estimates (results not shown). Lastly, the rather large proportions of missing observations in our variables of main interest (approximately 20% and mainly among participants born before 1966) might have led to a degree of bias towards the null, and this warrants cautious interpretation of our findings.

## 5. Conclusions

Our results suggested that birth order could influence childhood- and adult-onset T1D risk, irrespective of parental diabetes, migration background, birth weight, ever being breastfed and prematurity. The association was observed for only childhood-onset T1D in the whole cohort, but also for adult-onset T1D in a subgroup of participants born in the last few decades. These results supported the assumption that early exposure to more extensive microbiome might be a protective factor against the development of childhood- and also adult-onset T1D in relatively younger generations. These children were presumably exposed earlier to a more extensive microbiome from other children than single children or first-borns. No clear consistent associations between daycare attendance or C-section and T1D risk were observed in the cohort. However, taking into account the rising frequency of C-section worldwide and the previously concluded increased T1D risk linked to C-section, further research into the possible mechanisms of this association, especially the implication of bypassing labor, is imperative. In addition, after age 15, sex might be a determining factor in T1D manifestation, possibly due to hormonal changes in puberty. Our findings highlight the necessity to acknowledge sex differences in adult T1D risk assessment and diagnosis. Future studies investigating factors associated with adult-onset T1D and their underlying mechanisms are required for early risk detection and delaying the onset of clinical diabetes.

## Figures and Tables

**Table 1:** Description of the cohort by diabetes category based on our type 1 diabetes ascertainment algorithm.

	No Diabetes (n = 94,465)	Diabetes Type 1 (n = 371)	Diabetes Type 2 or Others (n = 6575)	Total (N = 101,411)
**Sex**				
- Male	43,264 (45.8%)	204 (57.6%)	3602 (54.6%)	47,070 (46.4%)
- Female	51,201 (54.2%)	167 (45.0%)	2973 (45.2%)	54,341 (53.6%)
**Caesarean delivery**				
- No	72,816 (77.1%)	259 (69.8%)	4290 (65.2%)	77,365 (76.3%)
- Yes	3489 (3.7%)	21 (5.7%)	105 (1.6%)	3615 (3.6%)
- Unknown	18,160 (19.2%)	91 (24.5%)	2180 (33.2%)	20,431 (20.1%)
**Birth order**				
- Only child	14,314 (15.2%)	69 (18.6%)	918 (14.0%)	15,301 (15.1%)
- First	24,765 (26.2%)	99 (26.7%)	1434 (21.8%)	26,298 (25.9%)
- Second	24,230 (25.6%)	81 (21.8%)	1299 (19.8%)	25,610 (25.3%)
- Third or more	16,116 (17.1%)	50 (13.5%)	1010 (15.4%)	17,176 (16.9%)
- Missing	15,040 (15.9%)	72 (19.4%)	1914 (29.1%)	17,026 (16.8%)
**Attended daycare**				
- No	25,408 (26.9%)	77 (20.8%)	2081 (31.7%)	27,566 (27.2%)
- Yes	54,091 (57.3%)	222 (59.8%)	2597 (39.5%)	56,910 (56.1%)
- Unknown	14,966 (15.8%)	72 (19.4%)	1897 (28.9%)	16,935 (16.7%)
**Birth year**				
- ≤1955	28,978 (30.7%)	73 (19.7%)	4142 (63.0%)	33,193 (32.7%)
- 1956–1965	27,341 (28.9%)	118 (31.8%)	1611 (24.5%)	29,070 (28.7%)
- 1966–1975	21,586 (22.9%)	97 (26.1%)	620 (9.4%)	22,303 (22.0%)
- 1976–1985	9299 (9.8%)	45 (12.1%)	175 (2.7%)	9519 (9.4%)
- ≥1986	7261 (7.7%)	38 (10.7%)	27 (0.4%)	7326 (7.2%)
**Paternal diabetes**				
- No	60,376 (63.9%)	195 (52.6%)	2615 (39.8%)	63,186 (62.3%)
- Yes, at age < 40 years	458 (0.5%)	12 (3.2%)	63 (1.0%)	533 (0.5%)
- Yes, at age ≥ 40 years/unknown	10,635 (11.3%)	66 (17.8%)	1201 (18.3%)	11,902 (11.7%)
- Unknown	22,996 (24.3%)	98 (26.4%)	2696 (41.0%)	25,790 (25.4%)
**Maternal diabetes**				
- No	66,019 (69.9%)	218 (58.8%)	2621 (39.9%)	68,858 (67.9%)
- Yes, at age < 40 years	403 (0.4%)	13 (3.5%)	94 (1.4%)	510 (0.5%)
- Yes, at age ≥ 40 years/unknown	11,119 (11.8%)	63 (17.0%)	1679 (25.5%)	12,861 (12.7%)
- Unknown	16,924 (17.9%)	77 (20.8%)	2181 (33.2%)	19,182 (18.9%)
**Migration background**				
- No migration background	79,517 (84.2%)	330 (88.9%)	5375 (81.8%)	85,222 (84.0%)
- Has migration background	14,936 (15.8%)	41 (11.1%)	1199 (18.2%)	16,176 (16.0%)
- Missing	12 (0.0%)	0 (0.0%)	1 (0.0%)	13 (0.0%)
**Premature birth** **(>4 weeks before due date)**				
- No	72,203 (76.4%)	270 (72.8%)	4142 (63.0%)	76,615 (75.5%)
- Yes	3179 (3.4%)	12 (3.2%)	183 (2.8%)	3374 (3.3%)
- Unknown	19,083 (20.2%)	89 (24.0%)	2250 (34.2%)	21,422 (21.1%)
**Birth weight**				
- Light	8018 (8.5%)	29 (7.8%)	511 (7.8%)	8558 (8.4%)
- Average	47,937 (50.7%)	176 (47.4%)	2482 (37.7%)	50,595 (49.9%)
- Heavy	8102 (8.6%)	34 (9.2%)	401 (6.1%)	8537 (8.4%)
- Unknown	30,408 (32.2%)	132 (35.6%)	3181 (48.4%)	33,721 (33.3%)
**Ever breastfed**				
- No	13,260 (14.0%)	60 (16.2%)	604 (9.2%)	13,924 (13.7%)
- Yes, >4 months	18,114 (19.2%)	66 (17.8%)	1047 (15.9%)	19,227 (19.0%)
- Yes, until 4 months	21,351 (22.6%)	79 (21.3%)	1200 (18.3%)	22,630 (22.3%)
- Unknown	41,740 (44.2%)	166 (44.7%)	3724 (56.6%)	45,630 (45.0%)
**BMI at age 18**				
- Underweight	8830 (9.3%)	24 (6.5%)	397 (6.0%)	9251 (9.1%)
- Normal weight	50,140 (53.1%)	173 (46.6%)	2519 (38.3%)	52,832 (52.1%)
- Overweight	5669 (6.0%)	31 (8.4%)	567 (16.1%)	6267 (6.2%)
- Obese	213 (0.2%)	3 (0.8%)	25 (0.7%)	241 (0.2%)
- Missing	29,613 (31.3%)	140 (37.7%)	3067 (46.6%)	32,820 (32.4%)

**Table 2:** Type 1 diabetes risk estimates from Cox regression models with all cases, onset age 0–15 years (n = 100) and onset age 16–40 years (n = 271).

Outcome: Type 1 Diabetes		n ^a^ (%)	Hazard Ratio (95% Confidence Interval)				
			Univariable	Multivariable Full Model ^b^	Multivariable Reduced Model ^b^		
Case Selection			Age at Diagnosis 0–40 Years			Age at Diagnosis 0–15 Years	Age at Diagnosis 16–40 Years
**Birth order**	Only child	15,301 (15.1)	1	1	1	1	1
	First	26,298 (25.9)	0.83 (0.61–1.12)	0.85 (0.63–1.17)	0.85 (0.62–1.16)	0.66 (0.38–1.15)	0.95 (0.65–1.39)
	Second	25,610 (25.3)	0.69 (0.50–0.96)	0.70 (0.50–0.96)	0.69 (0.50–0.96)	0.54 (0.30–0.95)	0.78 (0.52–1.15)
	≥Third	17,176 (16.9)	0.66 (0.46–0.95)	0.65 (0.45–0.94)	0.65 (0.45–0.94)	0.42 (0.20–0.87)	0.77 (0.50–1.19)
	Unknown	17,026 (16.8)	1.07 (0.77–1.49)	0.61 (0.13–2.91)	0.63 (0.13–3.01)	0.74 (0.03–20.23)	0.62 (0.11–3.66)
**C-section delivery**	No	77,365 (76.3)	1	1	1	1	1
	Yes	3615 (3.6)	1.51 (0.96–2.37)	1.32 (0.83–2.08)	1.35 (0.85–2.12)	1.68 (0.85–3.31)	1.13 (0.61–2.10)
	Unknown	20,431 (20.1)	1.48 (1.17–1.89)	1.46 (0.89–2.42)	1.43 (0.90–2.29)	0.97 (0.31–3.02)	1.58 (0.94–2.65)
**Attended daycare**	No	27,566 (27.2)	1	1	1	1	1
	Yes	56,910 (56.1)	1.12 (0.85–1.49)	1.05 (0.79–1.39)	1.04 (0.79–1.38)	0.84 (0.47–1.48)	1.11 (0.80–1.54)
	Unknown	16,935 (16.7)	1.50 (1.08–2.07)	1.46 (0.31–6.92)	1.42 (0.30–6.75)	1.71 (0.06–47.34)	1.36 (0.23–7.87)
**Sex**	Male	47,070 (46.4)	1	1	1	1	1
	Female	54,341 (53.6)	0.69 (0.56–0.85)	0.67 (0.54–0.83)	0.68 (0.55–0.84)	0.82 (0.55–1.22)	0.63 (0.50–0.81)

^a^ Number and percentage in the all-case analyses. ^b^ Full models adjusted for paternal diabetes, maternal diabetes, migration background, premature birth, birth weight and being breastfed history. Reduced models adjusted for paternal diabetes, maternal diabetes and migration background. All models were stratified by birth year.

## Data Availability

Restrictions apply to the availability of these data. Data was obtained from NAKO e.V. and are available at https://nako.de/ (accessed on 2 September 2019) with permission.

## Supplementary Materials

The following supporting information can be downloaded at: https://www.mdpi.com/article/10.3390/ijerph191710880/s1, Table S1: All-case type 1 diabetes risk estimates from Cox regression models among males and females, German National Cohort, 2014–2019; Table S2: Sensitivity analyses results for childhood-onset and adult-onset T1D risk, German National Cohort, 2014–2019; Table S3: Type 1 diabetes risk estimates grouped by birth year (cut-off year 1965) and onset age (cut-off age 15), German National Cohort, 2014–2019.
