# Supplementary material for: Birth Order, Caesarean Section, or Daycare Attendance in Relation to Child- and Adult-Onset Type 1 Diabetes: Results from the German National Cohort
Source: Int J Environ Res Public Health. 2022 Aug 31;19(17):10880. doi: 10.3390/ijerph191710880 (PMC9517906; doi:10.3390/ijerph191710880)
Supplement: Supplementary file 1 [file ijerph-19-10880-s001.zip › ijerph-1837989-supplementary.pdf]

Supplementary Table S1. All-case type 1 diabetes risk estimates from Cox regression models among males and females, German National Cohort, 2014 - 2019.

**Outcome: Type 1 diabetes**		**Male**				**Female**			
		**n (%)**	**HR (Univariable)**	**HR (Multivariable full[a])**	**HR (Multivariable reduced[a])**	**n (%)**	**HR (Univariable)**	**HR (Multivariable full[a])**	**HR (Multivariable reduced[a])**
**Birth order**	Only child	6949 (14.8)	1	1	1	8352 (15.4)	1	1	1
	First	12694 (27.0)	0.86 (0.58-1.29)	0.90 (0.60-1.35)	0.89 (0.60-1.33)	13604 (25.0)	0.74 (0.46-1.20)	0.80 (0.49-1.32)	0.80 (0.49-1.31)
	Second	12033 (25.6)	0.56 (0.36-0.86)	0.56 (0.36-0.88)	0.56 (0.36-0.87)	13577 (25.0)	0.86 (0.54-1.37)	0.90 (0.56-1.46)	0.90 (0.56-1.45)
	≥Third	8175 (17.4)	0.72 (0.46-1.16)	0.73 (0.46-1.17)	0.73 (0.46-1.17)	9001 (16.6)	0.53 (0.29-0.98)	0.54 (0.29-1.01)	0.53 (0.28-0.98)
	Unknown	7219 (15.3)	0.78 (0.48-1.26)	0.58 (0.07-5.09)	0.65 (0.07-5.73)	9807 (18.0)	1.48 (0.92-2.37)	0.64 (0.07-6.17)	0.57 (0.06-5.73)
**C-section delivery**	No	35568 (75.6)	1	1	1	41797 (76.9)	1	1	1
	Yes	1809 (3.8)	1.73 (0.99-3.02)	1.47 (0.83-2.59)	1.58 (0.90-2.76)	1806 (3.3)	1.12 (0.52-2.43)	1.04 (0.47-2.28)	1.03 (0.47-2.23)
	Unknown	9693 (20.6)	1.16 (0.82-1.63)	1.30 (0.72-2.33)	1.37 (0.79-2.37)	10738 (19.8)	1.94 (1.38-2.75)	2.07 (0.79-5.40)	1.67 (0.68-4.11)
**Attended day-care**	No	13207 (28.1)	1	1	1	14359 (26.4)	1	1	1
	Yes	26695 (56.7)	1.25 (0.87-1.80)	1.16 (0.80-1.68)	1.16 (0.80-1.67)	30215 (55.6)	0.95 (0.62-1.46)	0.89 (0.57-1.37)	0.89 (0.58-1.38)
	Unknown	7168 (15.2)	1.20 (0.75-1.91)	1.34 (0.16-11.54)	1.38 (0.16-12.08)	9767 (18.0)	1.84 (1.17-2.90)	1.59 (0.17-15.16)	1.33 (0.13-13.21)

[a] Full models adjusted for paternal diabetes, maternal diabetes, migration background, premature birth, birth weight and being breastfed history. Reduced models adjusted for paternal diabetes, maternal diabetes and migration background. All models were stratified by birth year.

Supplementary Table S2. Sensitivity analyses results for childhood-onset and adult-onset T1D risk, German National Cohort, 2014 - 2019.

**Outcome: Type 1 diabetes**		**(i) n[a] (%)**	**(i) HR[ac]**	**(i) n[b] (%)**	**(i) HR[bc]**	**(ii) n[a] (%)**	**(ii) HR[ac]**	**(ii) n[b] (%)**	**(ii) HR[bc]**
**Birth order**	Only child	14,383 (15.2)	1	14,359 (15.2)	1	15,301 (15.1)	1	15,279 (15.1)	1
	First	24,864 (26.2)	0.66 (0.38-1.15)	24,836 (26.2)	0.95 (0.65-1.39)	26,298 (25.9)	0.67 (0.38-1.18)	26,272 (25.9)	0.97 (0.68-1.38)
	Second	24,311 (25.6)	0.54 (0.30-0.95)	24,288 (25.6)	0.77 (0.52-1.14)	25,610 (25.3)	0.56 (0.31-1.01)	25,588 (25.3)	0.92 (0.64-1.31)
	≥ Third	16,166 (17.0)	0.42 (0.20-0.88)	16,155 (17.1)	0.76 (0.49-1.18)	17,176 (16.9)	0.47 (0.22-0.98)	17,165 (16.9)	0.75 (0.50-1.12)
	Unknown	15,112 (15.9)	0.80 (0.03-25.49)	15,098 (15.9)	0.64 (0.11-3.84)	17,026 (16.8)	0.75 (0.02-25.90)	17,014 (16.8)	0.63 (0.12-3.23)
**C-section delivery**	No	73,075 (77.1)	1	73,002 (77.1)	1	77,365 (76.3)	1	77,297 (76.3)	1
	Yes	3,510 (3.7)	1.66 (0.84-3.29)	3,500 (3.7)	1.12 (0.60-2.07)	3,615 (3.6)	1.80 (0.91-3.56)	3,605 (3.6)	0.77 (0.38-1.56)
	Unknown	18,251 (19.2)	0.99 (0.32-3.09)	18,234 (19.2)	1.61 (0.96-2.71)	20,431 (20.1)	1.03 (0.33-3.22)	20,416 (20.2)	1.62 (1.03-2.54)
**Attended day-care**	No	25,485 (26.9)	1	25,466 (26.9)	1	27,566 (27.2)	1	27,549 (27.2)	1
	Yes	54,313 (57.3)	0.84 (0.48-1.49)	54,246 (57.3)	1.12 (0.81-1.55)	56,910 (56.1)	0.88 (0.49-1.61)	56,846 (56.1)	0.91 (0.69-1.19)
	Unknown	15,038 (15.9)	1.56 (0.05-49.81)	15,024 (15.9)	1.31 (0.22-7.77)	16,935 (16.7)	1.63 (0.05-56.54)	16,923 (16.7)	1.08 (0.21-5.48)
**Sex**	Male	43,468 (45.8)	1	43,419 (45.8)	1	47,070 (46.4)	1	47,024 (46.4)	1
	Female	51,368 (54.2)	0.82 (0.55-1.21)	51,317 (54.2)	0.62 (0.48-0.79)	54,341 (53.6)	0.82 (0.54-1.23)	54,294 (53.6)	0.50 (0.40-0.63)

[a]Estimates for childhood-onset T1D. [b]Estimates for adult-onset T1D. [c] Multivariable model adjusted for paternal diabetes, maternal diabetes and migration background and stratified by birth year. (i) Participants with type 2 or other types of diabetes removed. (ii) Alternative T1D identification criteria applied were applied: 1) diagnosis age ≤50 years, 2) not first detected during pregnancy, 3) treated only with insulin within 1 year and 4) if therapy was unknown, then BMI at age 18 was <30 kg/m2 (not obese). Number of T1D cases = 423.

Supplementary Table S3. Type 1 diabetes risk estimates grouped by birth year (cut-off year 1965) and onset age (cut-off age 15), German National Cohort, 2014 - 2019.

**Outcome: Type 1 diabetes**		**Birth year ≤ 1965**				**Birth year > 1965**			
		**n (%)**	**HR (Multivariable reduced[a])**			**n (%)**	**HR (Multivariable reduced[a])**		
**Case selection**			**Age at diagnosis 0 – 40 years (n = 191)**	**Age at diagnosis 0 – 15 years (n = 38)**	**Age at diagnosis 16 – 40 years (n = 153)**		**Age at diagnosis 0 – 40 years (n = 180)**	**Age at diagnosis 0 – 15 years (n = 62)**	**Age at diagnosis 16 – 40 years (n= 118)**
**Birth order**	Only child	8556 (13.7)	1	1	1	6745 (17.2)	1	1	1
	First	15412 (24.8)	1.21 (0.75-1.95)	0.70 (0.28-1.76)	1.46 (0.83-2.57)	10886 (27.8)	0.67 (0.44-1.02)	0.69 (0.35-1.38)	0.65 (0.38-1.12)
	Second	14215 (22.8)	0.97 (0.59-1.61)	0.68 (0.27-1.74)	1.12 (0.61-2.03)	11395 (29.1)	0.55 (0.36-0.85)	0.48 (0.23-1.02)	0.59 (0.34-1.00)
	≥ Third	11068 (17.8)	0.72 (0.40-1.27)	0.27 (0.07-1.03)	0.94 (0.49-1.80)	6108 (15.6)	0.62 (0.38-1.02)	0.54 (0.22-1.28)	0.67 (0.37-1.22)
	Unknown	13012 (20.9)	0.35 (0.06-2.15)	0.76 (0.00-178.04)	0.35 (0.05-2.31)	4014 (10.3)	1.26 (0.21-7.44)	0.92 (0.02-34.91)	1.38 (0.20-9.32)
**C-section delivery**	No	46156 (74.1)	1	1	1	31209 (79.7)	1	1	1
	Yes	1065 (1.7)	0.94 (0.30-2.94)	2.73 (0.64-11.58)	0.40 (0.06-2.90)	2550 (6.5)	1.68 (1.02-2.78)	1.84 (0.86-3.94)	1.56 (0.80-3.04)
	Unknown	15042 (24.2)	1.38 (0.69-2.74)	1.91 (0.45-8.07)	1.26 (0.58-2.76)	5389 (13.8)	1.50 (0.79-2.82)	0.50 (0.08-3.13)	1.93 (0.97-3.85)
**Attended day-care**	No	23580 (37.9)	1	1	1	3986 (10.2)	1	1	1
	Yes	25701 (41.3)	1.23 (0.88-1.71)	1.11 (0.55-2.24)	1.27 (0.87-1.85)	31209 (79.7)	1.09 (0.65-1.83)	1.12 (0.44-2.89)	1.07 (0.58-1.99)
	Unknown	12982 (20.9)	3.65 (0.63-21.17)	1.04 (0.00 – 243.79)	4.49 (0.72-27.86)	3953 (10.1)	0.57 (0.09-3.52)	3.28 (0.08-130.85)	0.35 (0.05-2.45)
**Sex**	Male	29621 (47.6)	1	1	1	17449 (44.6)	1	1	1
	Female	32642 (52.4)	0.66 (0.49-0.88)	1.03 (0.54-1.95)	0.59 (0.43-0.82)	21699 (55.4)	0.73 (0.55-0.99)	0.77 (0.47-1.27)	0.71 (0.50-1.03)

[a] *Multivariable model adjusted for paternal diabetes, maternal diabetes and migration background.*
